# Direct and indirect cost of managing Alzheimer’s disease in the Islamic Republic of Iran

**Published:** 2019-01-05

**Authors:** Zahra Aajami, Abbas Kebriaeezadeh, Shekoufeh Nikfar

**Affiliations:** 1 Department of Pharmacoeconomics and Pharmaceutical Administration, School of Pharmacy, Tehran University of Medical Sciences, Tehran, Iran; 2 Pharmaceutical Management and Economics Research Center, Tehran University of Medical Sciences, Tehran, Iran

**Keywords:** Alzheimer Disease, Cost Analysis, Direct Cost, Indirect Expenditures

## Abstract

**Background:** Alzheimer’s disease (AD) affects a large number of adults annually all around the world. The monetary cost of this disorder is huge. This study aims to estimate the cost of AD in Iran by considering stages of disease.

**Methods:** A cross-sectional study was designed from July to December 2017 on 300 AD cases who referred to the Iran Alzheimer’s Association, Tehran, Iran. To calculate costs at different stages of disease, patients were assigned into three groups, based on the Mini-Mental State Exam (MMSE) score. A list of medicines’ prices and health care service costs were prepared. Health care services’ cost was acquired from the book of “Relative value units of health care services in Iran” and the price of medicines was extracted from "Iran’s medicine triple prices list". Patients’ medical records and face to face interview with their caregivers were used for data collection. The perspective of present research was societal.

**Results:** Annually, per person cost of AD in mild, moderate, and severe stages of disease were 434 United States dollars (USD), 1313 USD, and 2480 USD, respectively. Direct non-medical costs (DNMC) had the greatest share of total costs (near half of the whole costs) including 263 USD, 641 USD, and 1257 USD for mild, moderate, and severe stages, respectively.

**Conclusion:** The cost of AD in Iran is lower than the average cost of dementia in upper middle-income countries. In all stages, the biggest part of the cost is associated with patient care and nursing services because patients suffering from AD usually require specialized cares.

## Introduction

Dementia is a degenerative and irreversible neurologic impairment that involves content of consciousness.

About 10% of above 70 years old people have symptoms of dementia^1^ and in more than half of them it is caused by Alzheimer’s disease (AD); thus, AD is the most common cause of dementia.^2^

AD is characterized by cognitive impairment as well as psychological and behavioral findings and problems in the patient’s function and independence.^3^ Researchers have shown that AD is usually diagnosed 32 months after the onset of the disorder in the brain and these patients become institutionalized generally in 25 months. They always stay 44 months in this state. Hence, a course of AD will take averagely 101 months (near 8.5 years).^4^

At the moment, 46.8 million people are suffering from dementia and the figure will double every 20 years. One in 85 people will have AD by 2050.^5^^,^^6^

      The prevalence of AD in the elderly population is:

      People 65 to 85 years: 5%

      People ≥ 85 years: 20%-40%

      In nursing homes' residents: 50%^7^

There were 4.7 million AD cases (above 65 years old cases) in the United States (US) in 2010 and it will increase to 13.8 million in 2050.^4^ The incidence of the disease in the US has increased from 377000 cases in 1995 to 959000 cases in 2050.^8^ Over the next decades, the elderly population is going to increase much more rapidly than the total population in Iran like some other countries. Due to the increase of population’s aging process, the number of AD cases is increasing in our society. There are no exact statistics about the number of AD patients in Iran. Based on Gholamzadeh, et al. study, it is estimated that the prevalence of AD in people over 60 years old is 2.3%.^9^

Presently, there is no certain method for preventing or curing this disorder. For managing the symptoms of the disease, a cholinesterase inhibitor (donepezil, galantamine, and rivastigmine) is prescribed in mild and moderate AD and memantine is recommended in moderate AD cases who cannot use cholinesterase inhibitors and also in severe AD.^10^ The annual cost of care services for a patient with AD in severe stage is about 50000 US dollars (USD).^2^ The global cost of dementia increased from 604 billion USD in 2010 to 818 billion USD in 2015 and about half of this amount belongs to AD. This number is equivalent to 1% of the cumulative world gross domestic product (GDP), which presents the obvious and significant economic impact of this problem. It is predicted that the cost of dementia will increase to 1 and 2 trillion USD by 2018 and 2030, respectively.^11^ The monetary cost of dementia is like that of cardiovascular diseases (CVDs) and cancer.^12^ The annual cost of dementia in countries of the European Union is 189 billion USD.^13^ Table 1 shows the costs of dementia per person according to World Bank country classification (this classification is based on country’s income level) in 2015. As shown in table 1, there are significant differences between costs of patients with AD in high-income countries (HIC) and others. Although, the major part of AD cases (about 58%) are related to lower middle-income countries (LMIC), more than 90% of the total costs were incurred in HIC.^11^ These findings suggest that AD costs are high and this problem imposes large economic burden on the society. To date, no study has been published to estimate the direct and indirect cost (IC) of AD in Iran. Therefore, this research was carried out to evaluate the direct, indirect, and total cost of AD in Iran for helping policy makers for better allocation of resources. 

**Table 1:** Costs of dementia per person in 2015, based on World Bank country classification

**Type of country**	**Costs of dementia (USD)**
Low-income	1019
Lower middle-income	1560
Upper middle-income	5284
High-income	36669

## Materials and Methods

To estimate the costs of AD at different stages of the disease, a six-month plan was conducted to collect data from July to December 2017 on 300 AD cases referred to the Iran Alzheimer’s Association, Tehran, Iran. One hundred cases were enrolled from each stage of the disease (mild, moderate, and severe) in the research. Based on the Mini-Mental State Exam (MMSE) score, patients were categorized into three groups. These groups encompassed:

      Mild AD: MMSE score of 21-30

      Moderate AD: MMSE score of 11-20

      Severe AD: MMSE score of 0-10^14^^,^^15^

The MMSE score of each patient was calculated by neurologist in each visit and recorded in the patient’s file. 

The inclusion criteria were voluntary participation of over 65-year-old people.

Cases of early onset AD were excluded from the study. A list of medicines’ prices and health care services’ costs was prepared. This list was reviewed by five neurologists for editing and final appraisal. 

**Table 2:** Demographic characteristics of patients with Alzheimer’s disease (AD) in Iran

**Variables**		**Mild AD ** **(n = 100)**	**Moderate AD ** **(n = 100)**	**Severe AD ** **(n = 100)**
Age (year) (mean ± SD)		75.0 ± 5.0	80.0 ± 3.5	85.0 ±5.0
Gender	Men	52	50	42
	Women	48	50	58
Marital status	Married	62	52	44
	Divorced/widow	32	42	56
	Single	6	6	0
Education	Educated	64	48	32
	Illiterate	36	52	68
Average monthly income (USD) (mean ± SD)		208 ± 53	199 ± 32	186 ± 37
Number of children (mean ± SD)		4 ± 2	5 ± 2	5 ± 3
Having insurance coverage	Yes	96	96	100
	No	4	4	0

AD: Alzheimer’s disease; SD: Standard deviation

Healthcare services’ cost was acquired from the book of “Relative value units of health care services in Iran” and the price of medicines was extracted from "Iran’s medicine triple prices list". Patient’s medical records and face to face interview with their caregivers were used for data collection. The perspective of the present research was societal, so all costs were included in the study regardless of who the real payer was (medical insurances, patient’s family, and charity plans had covered the costs). The average annual cost was calculated for each stage of AD. The mentioned cost has three options: direct medical cost (DMC), direct non-medical cost (DNMC), IC. The steps for cost data gathering are described in the following:

DMC data were extracted simultaneously from patients' medical records at different stages of the disease and through face to face interviews with their caregivers.

 DMC includes all treatments and diagnostic interventions' cost as follows: expenditures of medicine, lab test, diagnostic and imaging services, visitation and consultation fees, and rehabilitation interventions (physiotherapy, occupational therapy, and...).

The average DMC for annual periods of treatment and care was calculated for each stage of AD. DNMC was computed according to the outcome of patient’s caregivers’ self-estimate questionnaire. The questionnaire measures non-medical expenditures for services like transportation, food, accommodation, nursing, and etc.

IC or productivity loss can be related to decreased productivity because of presenteeism or absenteeism. Presenteeism is a situation when people spend a lot of time at work even if they are ill or could take a holiday. To calculate IC, "Human Capital Approach" was applied.^16^

The information about IC was obtained by face-to-face interviews with patient’s caregivers using a self-report checklist, according to the number of workdays’ losses and mean daily wage.

In regard to the age of participants (65 years old and above), we calculated the IC only for patient’s caregivers. 

In order to perform international comparisons, all costs were converted from Iranian currency to USD by taking the exchange rate in January 2018 [48000 Iranian Rial (IRR) ~ 1 USD].^17^

Excel 2013 software was used for data analysis.

## Results

Demographic data are shown in table 2 for mild, moderate, and severe groups.

These data include: mean age, gender, marital status, education level, average monthly income, number of children, and insurance coverage status of whole groups.

Annually, DMC and its components for different stages of AD is shown in table 3. 

**Table 3:** Annual direct medical cost (DMC) of Alzheimer’s disease (AD) in Iran

**Type of cost**	**Mild AD**		**Moderate AD**		**Severe AD**	
	**Mean ± SD**	**%**	**Mean ± SD**	**%**	**Mean ± SD**	**%**
Medicine (USD)	62 ± 43	36.0	85 ± 43	39.0	110 ± 56	15.5
Lab test (USD)	6 ± 9	4.0	0	0	0	0
Diagnostic services (USD)	43 ± 39	25.0	0	0	20 ± 9	3.0
Consultation and visit (USD)	60 ± 17	35.0	58 ± 5	26.0	58 ± 5	8.5
Rehabilitation interventions (USD)	0	0	76 ± 36	35.0	514 ± 231	73.0
Total DMC (USD)	171 ± 81	100	219 ± 168	100	702 ± 172	100

SD: Standard deviation; AD: Alzheimer’s disease; DMC: Direct medical cost

**Table 4:** Annual direct non-medical cost (DNMC) of Alzheimer’s disease (AD) in Iran

**Type of cost**	**Mild AD**		**Moderate AD**		**Severe AD**	
	**Mean ± SD**	**%**	**Mean ± SD**	**%**	**Mean ± SD**	**%**
Transportation (USD)	98 ± 162	37.0	93 ± 16	14.5	47 ± 23	4.0
Meal (USD)	15 ± 24	6.0	26 ± 18	4.0	15 ± 30	1.0
Accommodation (USD)	10 ± 50	4.0	0	0	0	0
Nursing and home care (USD)	140 ± 532	53.0	507 ± 856	79.0	890 ± 1016	71.0
Equipment (pad,...) (USD)	0	0	15 ± 47	2.5	305 ± 120	24.0
Total DNMC (USD)	263 ± 559	100	641 ± 495	100	1257 ± 995	100

SD: Standard deviation; AD: Alzheimer’s disease; DNMC: Direct non-medical cost

According to this data, average DMC for mild, moderate, and severe stages of disease was 171 USD, 219 USD, and 702 USD, respectively, for a patient who suffers from AD.

In mild and moderate stages, the largest part of DMC was spent on medicine, while this option in severe stage belonged to rehabilitation interventions such as physiotherapy, occupational therapy, etc.

The DNMC in mild, moderate, and severe stages of AD was 263 USD, 641 USD, and 1257 USD, respectively (Table 4). In all the stages, the biggest part of the cost was used for nursing services and home caring for patients.

Table 5 shows all IC data of patients with AD in different stages. IC of the disease in mild, moderate, and severe stages of AD was 0 USD, 453 USD, and 521 USD, respectively. The minimum and maximum amounts of IC in moderate and severe phases were 0 USD and 2027 USD, respectively.

Based on the information in table 6, the largest part of the total cost of AD in all stages is DNMC.

**Figure 1:**
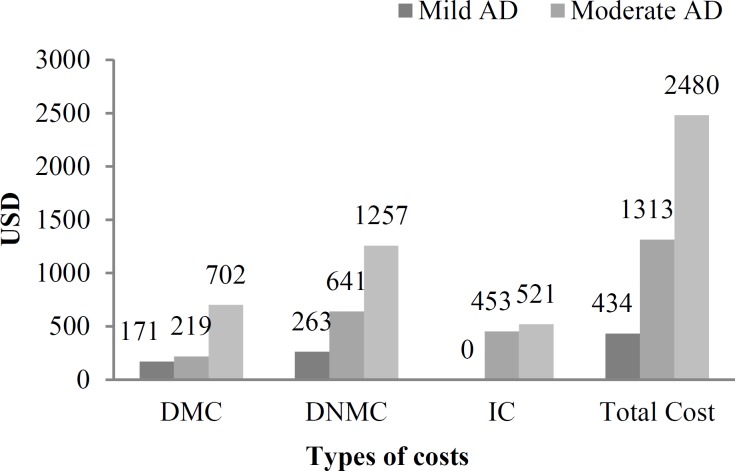
Comparison of direct medical cost (DMC), direct non-medical cost (DNMC), indirect cost (IC), and total cost in mild, moderate, and severe stages of Alzheimer’s disease (AD) in Iran

Also, it can be seen that there is a significant difference between total cost of mild and severe stages. Figure 1 compares the various costs in different stages of AD.

## Discussion

The current study is a comprehensive study performed to evaluate the direct cost and IC of AD type of dementia for the first time in Iran from a societal perspective. All costs (DMC, DNMC, and IC) for all stages of AD were calculated.

Annually, per person cost of AD in Iran for mild, moderate, and severe stages of AD was 434 USD, 1313 USD, and 2480 USD, respectively. Due to the average annual income of an Iranian urban household (about 6600 USD), AD cost seems huge.^18^ If an AD case is in the family, a significant part of the household income needs to be dedicated for managing this patient. If patient is covered by insurance system, only some parts of the DMC are paid by insurer and other costs (DNMC and IC) are borne by the family. There is no study calculating the cost of AD in Iran, so we cannot compare our findings with national figures, but it is possible to compare it with international data. 

**Table 5:** Annual indirect cost (IC) of Alzheimer’s disease (AD) in Iran

**Type of cost**	**Mild AD**		**Moderate AD**		**Severe AD**	
	**Mean ± SD**	**%**	**Mean ± SD**	**%**	**Mean ± SD**	**%**
Productivity loss (USD)	0	0	453 ± 673	100	521 ± 665	100
Total IC (USD)	0	0	453 ± 673	100	521 ± 665	100

SD: Standard deviation; AD: Alzheimer’s disease; IC: Indirect cost

**Table 6:** Types of costs in patients with Alzheimer’s disease (AD) in Iran

**Type of ** **cost**	**Mild AD**		**Moderate AD**				**Severe AD**	
	**Mean**	**%**		**Mean**	**%**	**Mean**		**%**
DMC (USD)	171	40		219	16	702		28
DNMC (USD)	263	60		641	49	1257		51
IC (USD)	0	0		453	35	521		21
Total cost (USD)	434	100		1313	100	2480		100

AD: Alzheimer’s disease; DMC: Direct medical cost; DNMC: Direct non-medical cost; IC: Indirect cost

The cost of AD treatment in Iran is lower than the average cost of dementia in upper middle-income countries (Table 1). The reason could be as a result of lower drug price in Iran that leads to lower DMC and fewer wage level resulting in smaller DNMC and IC in Iran.

Although in developed countries, more than 90% of patients with AD that need full-time care are delivered to nursing homes,^19^ in the present society the figure is far less and most of the time families are responsible for their patients with AD.

Researches have shown that direct cost (DMC and DNMC) includes about 86% of the total AD costs.

In all stages, the biggest part of the cost is associated with nursing services because patients that are suffering from moderate and severe AD need high-level specialized care.^19^^,^^20^ In the present analysis, the DNMC in mild, moderate, and severe stages of AD was 263 USD, 641 USD, and 1257 USD, respectively. According to the present findings, DNMC includes more than 50% of the total AD costs in Iran. In all stages, the biggest part of the DNMC was used for nursing services and home caring of patients like similar studies.

In this study, IC had the second and third position of contribution of the total costs in moderate and severe stages, respectively (35% and 21% of total cost, respectively). Since the whole cases were above 65 years old, IC alone was calculated using absenteeism calculation of caregivers (the Human Capital Approach). If interventions can slow down the progress of disease and shorten the stay time at the end stages, the heavy caring costs of AD will decrease.

Due to the inability of these patients, caregivers’ help is needed for data gathering and in most of the time they had no enough time or interest to answer the questions. So there was a big challenge in data gathering step.

Another limitation of this study was related to lack of sufficient data about IC and some components of DNMC in patients’ medical records. Such defects may lead to underestimation of the costs in some cases.

## Conclusion

In this study, we calculated all types of treatment costs of patients with AD in different stages. In all stages, DNMC and IC had the largest and smallest share of total costs, respectively. The biggest part of DNMC is related to the cost of nursing and home caring services. We recommend that further studies consider how to reduce these main actors of cost scenario share.

## Notes:

**How to cite this article:** Aajami Z, Kebriaeezadeh A, Nikfar S. Direct and indirect cost of managing Alzheimer’s disease in the Islamic Republic of Iran. Iran J Neurol 2019; 18(1): 7-12.

## References

[1] Alzheimer's Disease International World Alzheimer Report 2014: Dementia and Risk Reduction [Online]..

[2] Alzheimer's Disease Education &amp; Referral Center Alzheimer's disease [Online]..

[3] Jost BC, Grossberg GT (1995). The natural history of Alzheimer's disease: A brain bank study. J Am Geriatr Soc.

[4] Hebert LE, Weuve J, Scherr PA, Evans DA (2013). Alzheimer disease in the United States (2010-2050) estimated using the 2010 census. Neurology.

[5] Prince M, Bryce R, Ferri C (2011). World Alzheimer report 2011: The benefits of early diagnosis and intervention.

[6] Brookmeyer R, Johnson E, Ziegler-Graham K, Arrighi HM (2007). Forecasting the global burden of Alzheimer's disease. Alzheimers Dement.

[7] Zohari S, Khatouni S, Abed Saeidi ZH, Alavi Majd H, Yaghmaei F (2006). Problems of main caregivers of Alzheimer patients referring to Alzheimer association in Tehran. Faculty of Nursing of Midwifery Quarterly.

[8] Hebert LE, Beckett LA, Scherr PA, Evans DA (2001). Annual incidence of Alzheimer disease in the United States projected to the years 2000 through 2050. Alzheimer Dis Assoc Disord.

[9] Gholamzadeh S, Heshmati B, Mani A, Petramfar P, Baghery Z (2017). The prevalence of Alzheimer's disease; its risk and protective factors among the elderly population in Iran. Shiraz E-Med J.

[10] The National Institute for Health and Care Excellence Donepezil, Galantamine, Rivastigmine and Memantine for the treatment of Alzheimer's disease [Online]..

[11] Wimo A, Guerchet M, Ali GC, Wu YT, Prina AM, Winblad B (2017). The worldwide costs of dementia 2015 and comparisons with 2010. Alzheimers Dement.

[12] Hurd MD, Martorell P, Delavande A, Mullen KJ, Langa KM (2013). Monetary costs of dementia in the United States. N Engl J Med.

[13] Luengo-Fernandez R, Leal J, Gray AM (2011). Cost of dementia in the pre-enlargement countries of the European Union. J Alzheimers Dis.

[14] Folstein MF, Folstein SE, McHugh PR (1975). "Mini-mental state". A practical method for grading the cognitive state of patients for the clinician. J Psychiatr Res.

[15] Foroughan M, Jafari Z, Shirin Bayan P, Ghaem Magham Faraahani Z, Rahgozar M (2008). Validation of mini-mental state examination (MMSE) in the elderly population of Tehran. Adv Cogn Sci.

[16] Ganjali M, Baghfalaki T (2012). Bayesian analysis of unemploymentduration data in the presence of right andinterval censoring. Journal of Reliability and Statistical Studies.

[17] Central Bank of the Islamic Republic of Iran The Rate Currency Iran [Online]..

[18] Statistical Center of Iran Annual income of an Iranian urban household [Online]..

[19] Touchon J, Lachaine J, Beauchemin C, Granghaud A, Rive B, Bineau S (2014). The impact of memantine in combination with acetylcholinesterase inhibitors on admission of patients with Alzheimer's disease to nursing homes: Cost-effectiveness analysis in France. Eur J Health Econ.

[20] Suh GH (2009). Modeling the cost-effectiveness of galantamine for mild to moderately severe Alzheimer's disease in Korea. Value Health.

